# A meta-analysis on the potency of foot-and-mouth disease vaccines in different animal models

**DOI:** 10.1038/s41598-024-59755-4

**Published:** 2024-04-18

**Authors:** Jiao Jiao, Peng Wu

**Affiliations:** 1 College of Life Sciences, Shihezi University, Shihezi, China; 2 Ministry of Education Key Laboratory of Xinjiang Phytomedicine Resource Utilization, Shihezi, China; 3 Xinjiang Production and Construction Corps Key Laboratory of Oasis Town and Mountain-Basin System Ecology, Shihezi, China

**Keywords:** Vaccines, Pigs, Foot and mouth disease, Meta-analysis, Immunology, Infectious diseases, Viral infection

## Abstract

Whether mice can be used as a foot-and-mouth disease (FMD) model has been debated for a long time. However, the major histocompatibility complex between pigs and mice is very different. In this study, the protective effects of FMD vaccines in different animal models were analyzed by a meta-analysis. The databases PubMed, China Knowledge Infrastructure, EMBASE, and Baidu Academic were searched. For this purpose, we evaluated evidence from 14 studies that included 869 animals with FMD vaccines. A random effects model was used to combine effects using Review Manager 5.4 software. A forest plot showed that the protective effects in pigs were statistically non-significant from those in mice [MH = 0.56, 90% CI (0.20, 1.53), P = 0.26]. The protective effects in pigs were also statistically non-significant from those in guinea pigs [MH = 0.67, 95% CI (0.37, 1.21), P = 0.18] and suckling mice [MH = 1.70, 95% CI (0.10, 28.08), P = 0.71]. Non-inferiority test could provide a hypothesis that the models (mice, suckling mice and guinea pigs) could replace pigs as FMDV vaccine models to test the protective effect of the vaccine. Strict standard procedures should be established to promote the assumption that mice and guinea pigs should replace pigs in vaccine evaluation.

## Introduction

Foot and mouth disease virus (FMDV) belongs to *Picornaviridae*, which is a single-stranded positive-sense RNA virus of the genus *Aftab*^1^. FMD is listed among the highly contagious diseases in animals and is endemic in Africa, most of Asia, the Middle East, and parts of South America^2^. FMD endemic regions contain three-quarters of the world’s FMD-susceptible livestock and most of the world’s poorer livestock keepers^3^.

Vaccines play an important role in controlling FMD^4^. There are serological tests, virus neutralization tests, and enzyme-linked immune sorbent assay (ELISA) methods to evaluate the immune efficacy of FMD vaccines, but the most reliable method is the in vivo protection test to determine the 50% protective dose or the protective rate of systemic hoof infection ^5^. Efficacy tests of other target animals (such as sheep, goats, or buffaloes) and the use of different methods have not been standardized (OIE Manual Terrestrial)^6^. It would be very valuable to verify the expected protection rate of a vaccine with cattle and to estimate the possibility that cattle can resist 10,000 infective doses after one vaccination^7,8^. However, it is difficult to use cattle when evaluating the efficacy of a vaccine. Cattle need many people for their care, they are dangerous, and they are expensive. Particularly in the exploratory stage of vaccine research, the laboratory stage, a new evaluation model would be beneficial for the development of new vaccines^6^. Different animal models are usually used in the research of FMDV vaccines^9^. The models used to evaluate laboratory vaccine effects include guinea pigs, mice, and suckling mice^10^. When studying the protective efficacy of vaccines, mice and guinea pigs are often used as substitutes for pigs^11^. The use of mice and guinea pigs simplifies the experimental process^12^. As a model animal, mice have incomparable advantages^13^, such as simple operations, and a large number of reports with considerable data regarding mice as FMD vaccine models^14,15^. However, the major histocompatibility complex (MHC) of mice and guinea pigs is very different from that of pigs^16,17^, and some animal models may not be appropriate for the vaccine evaluation of pigs^18,19^.

At present, there are no related literature reports on the correlation between the results of mice and pigs for FMDV vaccines. The ultimate goal of this meta-analysis study was to explore the rationality of replacing large animals with small animal models for vaccine testing. A meta-analysis can increase the credibility of the conclusion and support the analysis of controversial arguments^20^. A meta-analysis increases the statistical efficiency that a single experiment does not have, and has guiding significance for follow-up clinical experiments^21^. We summarized previous experimental data by employing statistical methods to avoid using and injuring a large number of animals. To clarify the possibility of using different animal models instead of pigs for FMD potency studies, a meta-analysis was performed in the present study.

## Materials and methods

### Literature search strategy

For this systematic review with meta-analysis (JJ and PW) searched literature published from January 1995 to August 2023. The databases PubMed, China Knowledge Infrastructure (CNKI), EMBASE, and Baidu Academic were used to search for FMDV models. The keywords were as follows: “FMDV, “mice,” “guinea pigs,” “pig or swine,” and “vaccine.” Efforts were made to include relevant gray literature, but none was found.

### Inclusion and exclusion criteria

The inclusion standards were as follows: ① published Chinese and English literature on FMDV immune animal models; ② studies that included more than two animal models; ③ documents that included challenge potency (direct potency, not only serology) studies with FMDV; ④ the number of animals in the experiment was reported accurately in the literature; and ⑤ published studies and gray literature dated from January 1995 to August 2023.

The exclusion standards were as follows: ① systematic reviews without animal experiments; ② FMD models were not included; ③ when other reports provide the same data, the latest published data will be taken into account; and ④ the literature did not include a clear number of experimental animals.

### Data extraction

Two researchers performed preliminary screening by reading titles and abstracts. Then, we read the full text and selected documents for further analysis according to the inclusion and exclusion criteria. Any differences of opinion were settled through discussion. Data were extracted independently and entered into a specially designed data extraction table. The extracted data included the first author, publication time, number of animals, number of protected animals, and other similar information. "Event" referred to the number of protected animals. The database was built using Microsoft Office Home and Student 2021 software.

### Statistical analysis

Meta-analyses were performed using Review Manager 5.4 software (RevMan 5.4) provided by the Cochrane Collaboration. Statistical heterogeneity was quantified using the tau parameter that estimates the dispersion of the true treatment effects across the studies. Combined effect sizes and 95% confidence intervals (CI) were calculated using a random-effects model. The random-effects model used built-in modules in RevMan 5.4 software. The Mantel–Haenszel method was used to analyze the combination of effects. A funnel plot was used for the visual (and fully subjective) investigation of possible small-study effects. For data analysis, the groups were divided by different animal models. To study the protective effects of the different models, we conducted an analysis comparing the swine group with the control group. We conducted a non-inferiority analysis of the data. Non-inferiority was investigated by JMP software. The non-inferiority boundary value was set to 0.5. We used X to fit Y for non-inferiority tests. Through the relationship between the upper and lower limits of 90% difference and the boundary value, the result could be directly judged.

## Results

### Identified study reports

**Figure 1:**
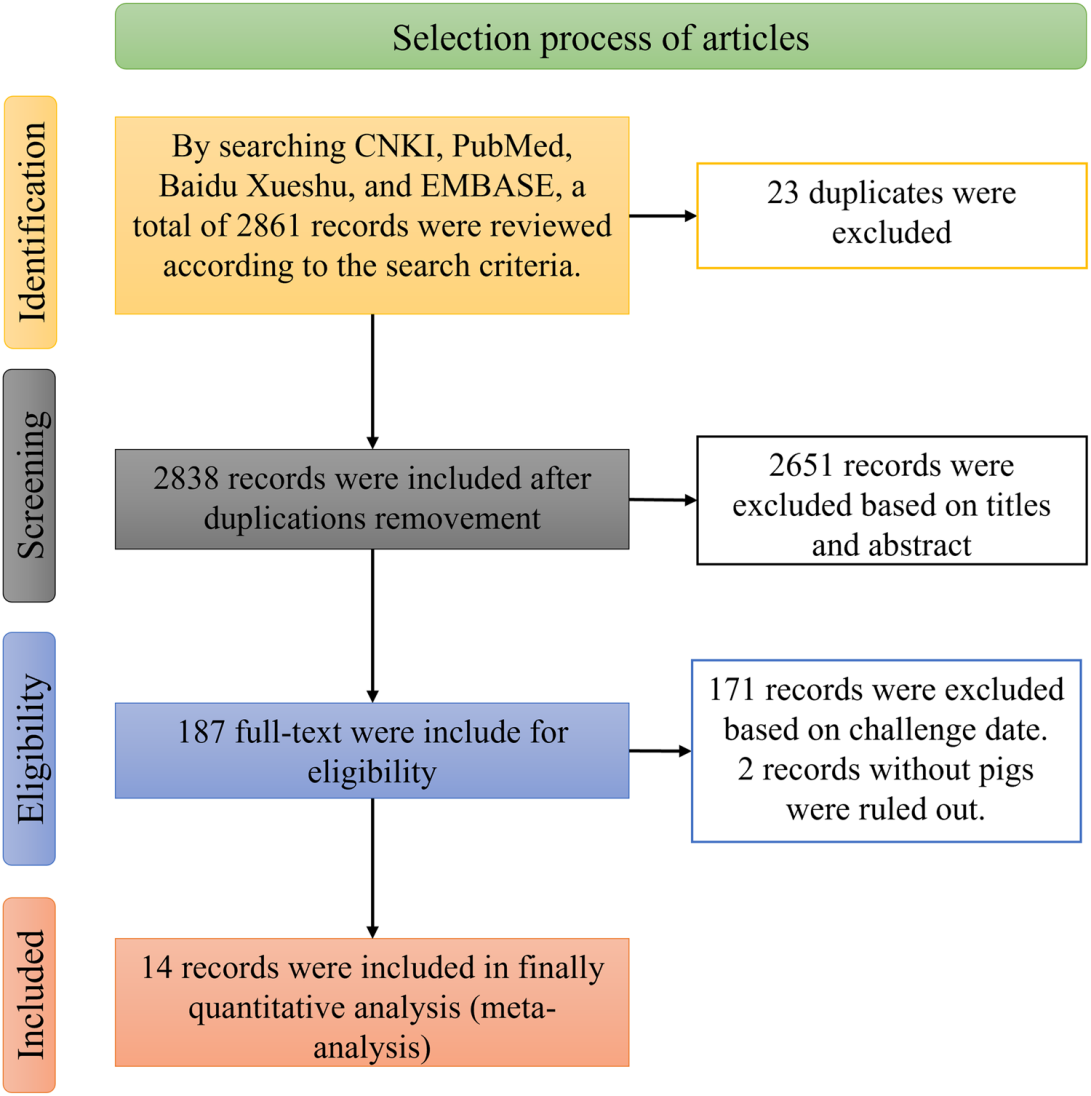
Flowchart of included and excluded trials.

The literature was searched and screened (Fig. 1). A total of 2861 literature reports were retrieved from PubMed, CNKI, EMBASE, and Baidu Academic. After removing 23 duplicate articles and reading titles and abstracts, 189 articles met the inclusion criteria. A total of 14 articles were included in the meta-analysis.

### Characteristics of the reports

Table 1 shows the features of the selected studies. A total of 869 animals were included in the meta-analysis. The animals in this research included mice, guinea pigs, and pigs. The research period was from 1997 to 2023, and it included 14 studies (Table 1).

**Table 1:** Characteristics and summary findings of the selected studies.

	Author	Year	Treatment1	Event1	n1	Treatment2	Event2	n2	Treatment3	Event3	n3	
1	Gisselle N. Medina	2023	Swine	19	28	Mice	12	12				^22^
2	Ji-Hyeon Hwang	2021	Swine	2	4	Mice	23	30				^23^
3	Hyundong Jo	2021	Swine	12	16	Mice	39	50				^24^
4	Yanmei Dong	2015	Guinea pigs	34	60	Swine	8	15				^15^
5	Teresa Rodrı guez-Calvo	2010	Mice	37	37	Swine	12	14				^14^
6	Carolina Cubillos	2008	Guinea pigs	10	10	Swine	10	10				^11^
7	Houhui Song	2005	Swine	8	10	Mice	59	90				^25^
8	Jun	2005	Mice	116	134	Swine	2	2				
9	Guangjin Li	2004	Swine	15	20	Guinea pigs	12	12				^26^
10	Ligang Wu	2003	Swine	3	3	Suckling mice	15	20	Guinea pigs	29	38	^27^
11	EWC chan	2001	Suckling mice	12	12	Swine	12	15				^28^
12	MA Kuprianova	2000	Swine	3	6	Guinea pigs	24	47				^29^
13	Quanxing Xu	1998	guinea pigs	69	77	Swine	52	65				
14	Yongjin You	1997	swine	7	12	Guinea pigs	12	20				

### Meta-analysis

**Figure 2:**
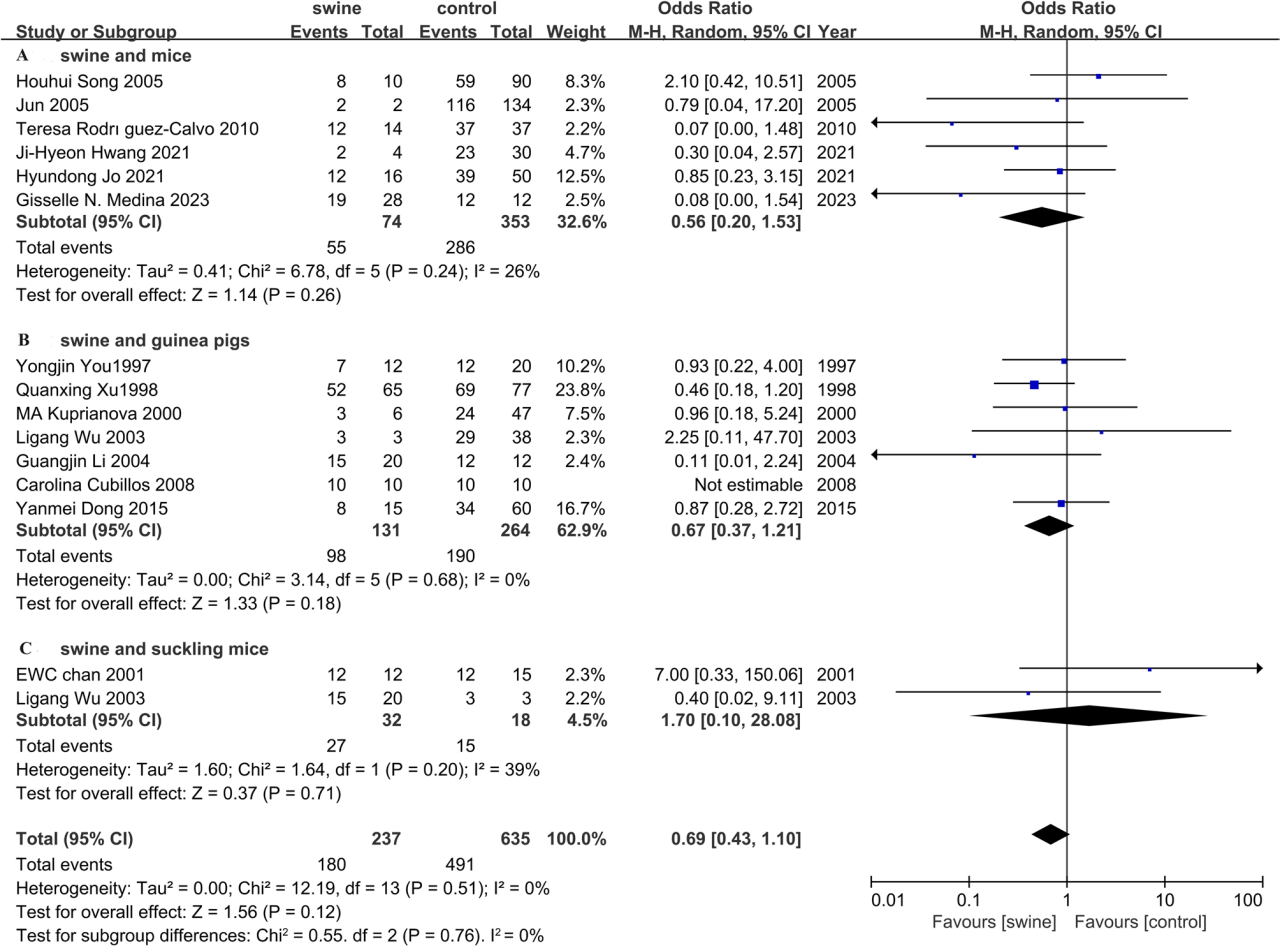
Forest plot.

The results of the forest plot showed statistically non-significant differences between different animal models (mice, suckling mice, and guinea pigs) and swine with FMDV [MH = 0.69, 95% CI (0.43, 1.10), *P* = 0.12] (Fig. 2). The forest plot showed that the protective effects in pigs were statistically non-significant from those in mice [MH = 0.56, 95% CI (0.20, 1.53), *P* = 0.26] (Fig. 2A).

The results showed that the protective effects of guinea pigs were statistically non-significant from those of pigs [MH = 0.67, 95% CI (0.37, 1.21), *P* = 0.18] (Fig. 2B). There were statistically non-significant differences between swine and suckling mice [MH = 1.70, 95% CI (0.10, 28.08), *P* = 0.71] (Fig. 2C). At present, there were only two articles on the relationship between swine and suckling mice.

The forest plot clearly showed serious statistical heterogeneity with study results pointing to different directions. The result of I^2^ was not consistent with the forest map. Although the value of I^2^ was small, it also had serious statistical heterogeneity. There were few relevant literature reports because the extraction standard of the meta-analysis required that two controlled experiments must appear in the same article.

**Figure 3:**
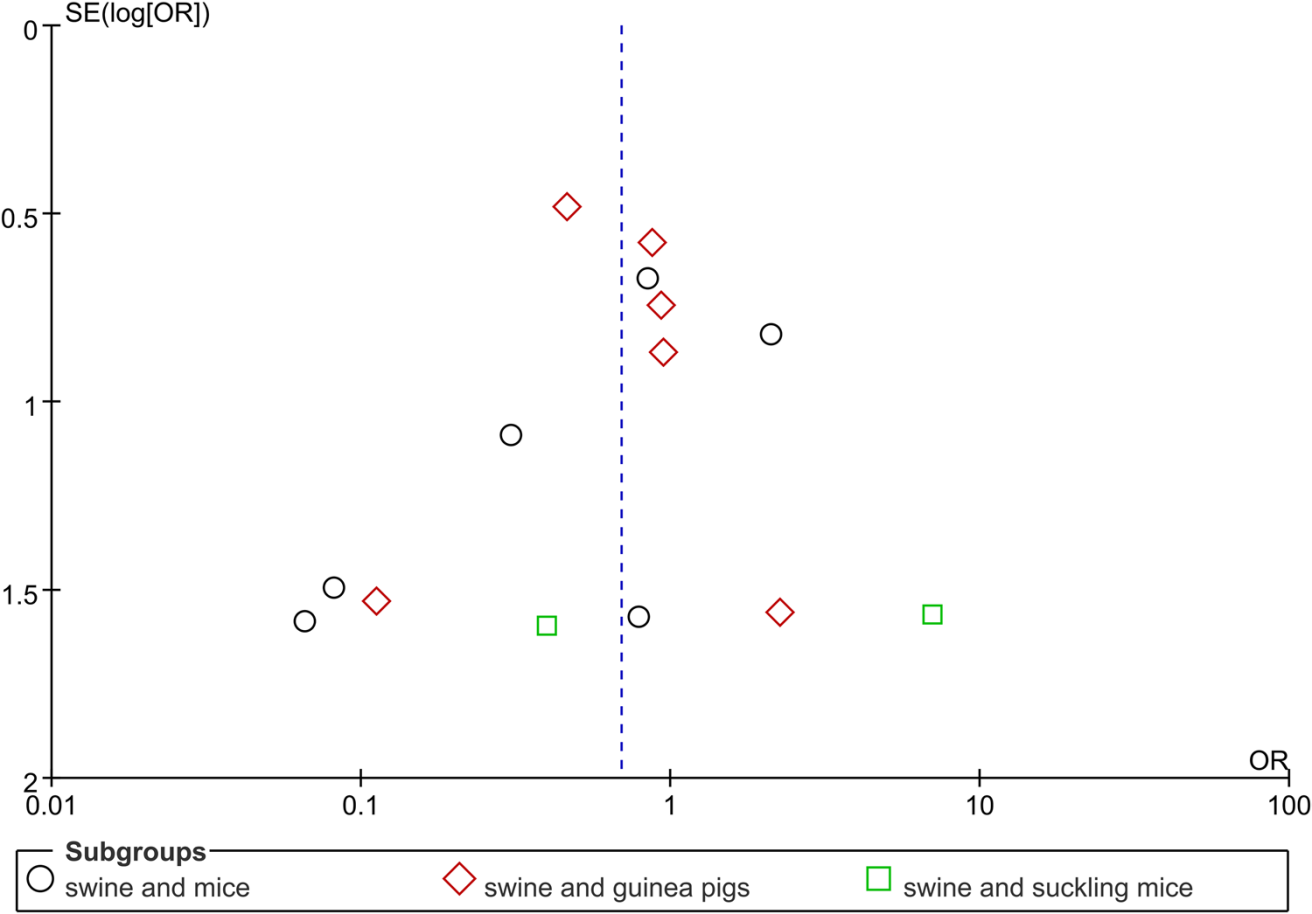
Funnel plot.

A funnel plot was used for the visual (and fully subjective) investigation of possible small-study effects (Fig. 3). Overall, the plot resembled a funnel chart. However, the funnel charts of the three subgroups were not ideal by themselves. The reason may be that there were too few studies included in the subgroups, and the subgroups were not suitable for use in funnel charts.

**Figure 4:**
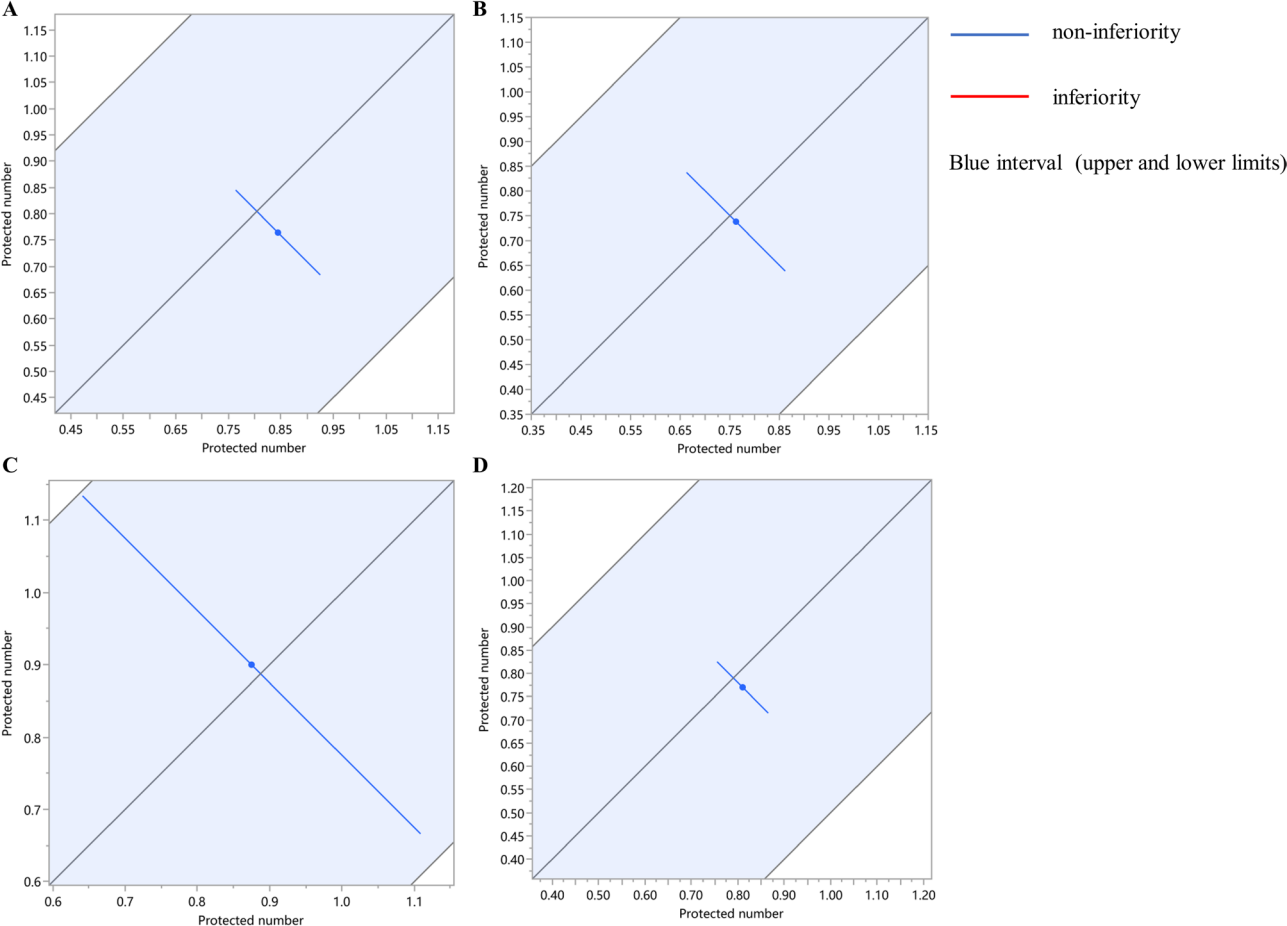
Non-inferiority plot. (**A**) Non-inferiority was tested with mice and pigs. (**B**) Non-inferiority was tested with guinea pigs and pigs. (**C**) Non-inferiority was tested with suckling mice and pigs. (**D**) Mice, guinea pigs, and suckling mice was made non-inferiority test to pigs. When the blue line (90% confidence interval) is included in the blue interval (upper and lower bounds), a non-inferiority conclusion could be drawn. When the red line (90% confidence interval) is not included in the blue interval (upper and lower bounds), a non-inferiority conclusion cannot be drawn.

Non-inferiority test could provide a hypothesis that the models (mice, suckling mice and guinea pigs) could replace pigs as FMDV vaccine models to test the protective effect of the vaccine (Fig.4). Through meta-analysis, we found that there was some heterogeneity in this study (Fig. 2). Even though the null hypothesis was rejected in all tests, the results should be interpreted with caution due to the substantial statistical heterogeneity observed in the forest plot (Fig. 4).

## Discussion

FMD is a highly contagious and destructive virus^30^. There are very strict restrictions on FMD experiments, and the requirements for the laboratory are also very high^31^. These existing conditions restrict the development of experiments and the acquisition of data on FMD. A meta-analysis assumes that the processed data are normally distributed^32^. In principle, the data should conform to a normal distribution^32^. The occurrence of zero events has a great impact on META-analysis^33^. We have tried our best to collect appropriate data.

As model animals, mice have the advantages of clean genetic backgrounds, easy breeding, and simple acquisition^14,15^. Compared with pigs, mice are more accessible^12^. It is easy to administer vaccines and drugs to mice by injection^13^. The injection dose for mice is less than that for pigs, which is more suitable for preliminary research. However, the MHC of mice and pigs is different^16,17^. Antibodies against the same antigen are also different^18,19^. The forest plot showed that the protective effects on pigs were statistically non-significant from those of mice [MH = 0.56, 95% CI (0.20, 1.53), *P* = 0.26] (Fig. 2A).

We innovatively compared different models, which also involved heterogeneity of methods^34^. Although clinical and methodological heterogeneity was always present, in many studies, mice and guinea pigs were used instead of pigs to evaluate vaccine effects. Although different methods increase heterogeneity, a scientific selection of indicators can reduce heterogeneity as much as possible, so that the results of the two models tend to be similar. We made a direct comparison between mice and pigs, guinea pigs and pigs, and suckling mice and pigs. There was no comparison between mice, suckling mice, and guinea pigs directly. Network meta-analysis (NMA) may help to directly compare different models^35^. To visually investigate small-study effects in NMA, Chaimani and colleagues developed a tool^36,37^. Mavridis et al. extended the Copa selection model for publication bias to NMA^38^. A transitivity assumption is the cornerstone of NMA; it posits that the comparisons do not differ beyond the interventions compared^39^. However, the different models we studied were not applicable to NMA. We chose RevMan to perform a traditional meta-analysis.

There are some limitations in this study. There are many guidelines for performing a meta-analysis^40^. A meta-analysis has comprehensive and objective advantages, including data integration^41^. There may be some heterogeneity and deviations in any research^42^. First, the inconsistent dosages administered to animals may affect the experimental results, leading to heterogeneity. Second, a funnel plot was used for the visual (and fully subjective) investigation of possible small-study effects. In this study, reducing the occurrence of deviations was of prime importance. Some of the retrieved data may be ignored, such as data in different languages, from different databases, and using different keywords. Inclusion and exclusion criteria may also lead to bias, and deviations may also appear at different steps in the process. However, according to the funnel chart, the bias was within the acceptable range.

In this study, to the best of our knowledge, a systematic review and meta-analysis were used for the first time to analyze the immune effects of different FMD animal models. Non-inferiority test can provide a hypothesis that the models (mice, suckling mice and guinea pigs) can replace pigs as FMDV vaccine models to test the vaccine protection effect. Reasonable selection of animal models can not only reduce the use of experimental animals but also promote the evaluation of vaccine effects, thus improving the protective effects of the vaccine. It is very valuable to compare the effects on a small animal model with the effects on pigs. Our experiment results will improve the rationality of the model. Furthermore, the cost of vaccine research and development is reduced. Animal models have accelerated the speed of vaccine development. Whether the results of the model can be used as an OIE standard still needs further research and efforts.

## Conclusion

In conclusion, non-inferiority test could provide a hypothesis that the models (mice, suckling mice and guinea pigs) could replace pigs as FMDV vaccine models to test the protective effect of the vaccine. Strict standard procedures should be established to promote the assumption that mice and guinea pigs should replace pigs in vaccine evaluation.

## Data Availability

All data generated or analyzed during this study were included in this published article.
